# Lateral ventricle pleomorphic xanthoastrocytoma concurrent with Dandy-Walker complex: A case report

**DOI:** 10.1097/MD.0000000000030492

**Published:** 2022-09-09

**Authors:** Tian-Fei Luo, Yu-Bo Wang, Dan-Hua Wang, Shuang Zhan, Shuang-Lin Deng

**Affiliations:** a Department of Neurology, First Hospital of Jilin University, Chang Chun, China; b Department of Oncological Neurosurgery, First Hospital of Jilin University, Chang Chun, China; c Department of Pathology, First Hospital of Jilin University, Chang Chun, China.

**Keywords:** congenital malformation, Dandy-Walker complex, pleomorphic xanthoastrocytoma

## Abstract

Dandy-Walker complex and pleomorphic xanthoastrocytomas are both rare disease entities that typically manifest early in life and are associated with congenital etiological factors. Dandy-Walker complex is a cerebellar malformation associated with a series of anatomical changes. The disease onset is usually at birth or during infancy. Late onset in adulthood is uncommon. Pleomorphic xanthoastrocytoma is a rare WHO grade II astrocytic tumor affecting mainly young adults. Concomitant occurrence of Dandy-Walker complex and pleomorphic xanthoastrocytoma has not been previously reported.

**Patient concerns and diagnosis:**

A 30-year-old woman with a previous history of unconfirmed resected lateral ventricle meningioma presented with severe headache for 1 day. Imaging examination revealed a mass in the right lateral ventricle with heterogeneous signal patterns, changes in the posterior fossa corresponding to a Dandy-Walker variant, and mild hydrocephalus.

**Interventions and outcomes:**

Surgical complete resection of the mass was achieved. postoperative histopathological examination confirmed WHO grade II pleomorphic xanthoastrocytoma. Three years postsurgery, ventriculoperitoneal shunt was performed due to worsening of hydrocephalus. The patient has since remained symptom-free.

**Conclusion:**

This is the first report of concomitant occurrence of Dandy-Walker complex and pleomorphic xanthoastrocytoma. The association of neurological congenital malformation with intracranial neoplasms may be multifactorial, with underlying role of genetic mutations or chromosome alterations.

## 1. Introduction

Dandy-Walker complex is an uncommon congenital cerebellar malformation with a reported incidence of 1/25,000 to 1/35,000 births. It is typically characterized by a triad of features, that is, cerebellar dysgenesis, cystic dilation of the fourth ventricle, and an enlarged posterior fossa.^[1]^ Despite the heterogeneous clinical manifestations, most cases are diagnosed in early infancy or childhood. Reports of delayed diagnoses in adulthood are rare, and some of these cases are incidentally diagnosed during screening for other pathologies.

Pleomorphic xanthoastrocytoma (PXA) is a relatively rare glial neoplasm that accounts for <1% of all intracranial neoplasms. The condition typically affects young adults. The majority of PXA occur as supratentorial lesions. The most common site of occurrence of PXA is temporal lobe followed by parietal and occipital lobes.^[2]^ Owing to the tendency for cortical involvement, seizure is one of the most prominent manifestations of these tumors, which facilitates early detection.^[3]^ On the contrary, deep-seated PXA is rarer, and there are very few reports of PXAs involving the ventricles.

Concomitant occurrence of Dandy-Walker complex and intracranial neoplasms is extremely rare, and to the best of our knowledge, there are no previous reports of PXA concurrent with Dandy-Walker complex. Herein, we report a case of lateral ventricle PXA with concomitant Dandy-Walker complex treated with tumor resection and ventriculoperitoneal shunt because of subsequent worsening of hydrocephalus.

## 2. Case report

A 30-year-old woman was admitted to our department for sudden onset of severe headache for the last 1 day. Upon admission, the patient had normal appearance and head size. Physical examination was unremarkable with normal motor and sensory function, coordination, cognitive and verbal function, and memory function. The patient recalled a previous surgery for lateral ventricle meningioma at a local hospital 10 years ago; however, the medical records were not available for confirmation of the diagnosis. Laboratory investigations were normal. The patient denied any history of epilepsy, and her family history was unremarkable.

**Figure 1.**
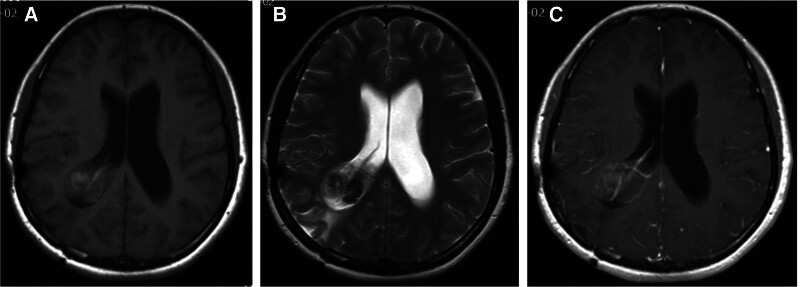
MRI images showing the PXA in the lateral ventricle. Axial T1W1 sequence shows a hypointense right lateral ventricle mass with patchy hyperintense signals (A). The mass shows mixed signals on T2WI sequence (B). Heterogeneous enhancement is seen on T1WI contrast-enhanced sequence (C). PXA = pleomorphic xanthoastrocytoma.

**Figure 2.**
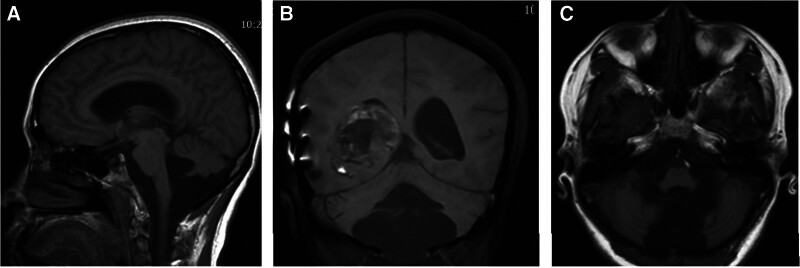
Posterior fossa MRI sections showing Dandy-Walker variant. Imaging of the posterior fossa showing increased retrocerebellar cerebrospinal fluid in the sagittal plane (A), cerebellar vermis hypoplasia with lateral ventricle mass in the coronal plane (B), and enlargement of the cerebellomedullary cistern and the fourth ventricle (A and C). The height of the torcula is normal (A).

MRI study revealed signal changes in the skull and the adjacent temporal-occipital lobe indicating a previous surgery. A 4.2 cm × 3.7 cm × 3.4 cm lesion containing cysts was observed at the atrium of the right lateral ventricle. On TIWI sequence, the lesion appeared hypointense with patchy areas of hyperintense signals. T2W1 sequence showed mixed signals and dark-fluid signals. On contrast-enhanced sequence, the lesion showed heterogeneous patchy enhancement sparing the intralesional cysts (Fig. 1). Imaging of the posterior fossa showed cerebellar vermis hypoplasia, enlargement of the cerebellomedullary cistern and the fourth ventricle, and increased retrocerebellar cerebrospinal fluid. The height of the torcula was normal. Axial images revealed a communication between the fourth ventricle and the posterior fossa fluid (Fig. 2). There was mild enlargement of the lateral ventricles. The patient was diagnosed with Dandy-Walker variant with concomitant lateral ventricle tumor and mild hydrocephalus.

**Figure 3.**
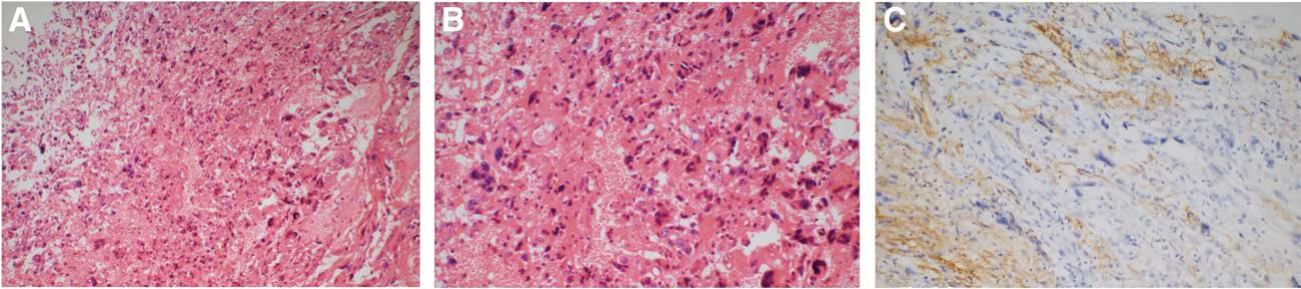
Histopathological examination of the resected PXA. Hematoxylin and eosin-stained section (A, ×100; B, ×200) showing pleomorphic spindle-shaped tumor cells with multinuclear giant cells. Positivity for GFAP immunostaining is observed (C, ×200). PXA = pleomorphic xanthoastrocytoma.

Surgical resection of the tumor was performed through the entry point of the previous surgery. Approximately 2 cm beneath the cortical surface, the tumor was reddish in color and had soft texture with rich blood supply and intratumoral apoplexy. The peripheral part of the tumor was relatively clear but was mildly adhered to the ventricular wall. Complete resection of the tumor was achieved. Histopathological examination confirmed WHO grade II PXA, with positive staining for CD34, oligodendrocyte transcription factor 2, neuron-specific enolase, CD56, vimentin but negative staining for TIF-1, EMA, and CD68 (Fig. 3). Ki-67 labeling index (marker of proliferation) was 5%, the P53 fraction was 40%, and the fraction of MGMT immune-positive tumor cells was 60%. The postoperative course was uneventful.

Three years postsurgery, the patient was readmitted to our department for recurrence of gradually worsening headache. Imaging study showed worsening of hydrocephalus. Ventriculoperitoneal shunt was performed which alleviated the symptoms. The patient has remained recurrence-free as of the most recent follow-up.

## 3. Discussion and literature review

The term Dandy-Walker malformation (DWM) was first used by Dandy and Blackfan in 1914 to describe the anatomical alterations in the cerebellum and the fourth ventricle. The term Dandy-Walker syndrome was later proposed in 1954, and the term has since been interchangeably used with DWM in many scenarios.^[4]^ However, there is no clear consensus on the precise diagnostic criteria, and the nomenclatures have constantly evolved with inclusion or specification of related conditions, which has added to the complexity of the condition. Currently, the scope of this disease has been expanded to include a continuum of posterior fossa cystic malformations, including the traditional malformation together with Dandy-Walker variant and maga cisterna magna, which is referred to as the term Dandy-Walker complex or Dandy-Walker continuum. To date, the pathogenetic mechanism of this disease is not well characterized. The majority of the cases are sporadic and a specific etiology is identifiable only in a small proportion of patients; the reported etiologies include abnormalities of chromosomes 3, 9, 13, 18^[5]^ and mutations of *FOXC1*, *ZIC1*, and *ZIC4* genes.^[6,7]^

The overall incidence of DWM is low. According to a recent epidemiological study, the estimated prevalence of DWM in Europe is 6.79 per 100,000 births, with live birth reported in 39.2% cases.^[8]^ As a severe malformation, the majority of the cases are either identified in the early childhood or during infancy/in utero. DWM may cause a wide range of signs and symptoms related to hydrocephalus, and cerebellar and cranial nerve dysfunction. These symptoms usually appear in the first year of life, hence early diagnosis is common.^[9]^ Delayed diagnosis until adulthood is rare. The cases diagnosed in adulthood usually harbored mild symptoms or were asymptomatic until incidental detection of the condition. One of the largest case series focusing on incidental detection of DWM in asymptomatic patients included 3 adults; of these 2 patients had mild traumatic injury and 1 had trigeminal neuralgia, all unrelated to the malformation.^[10]^ Adult patients with late onset of symptoms related to the malformation have also been reported; the symptoms may include neural dysfunction manifesting as tremor, sensorimotor disorders, and in cases of Dandy-Walker variants, neurocognitive disorders such as psychosis and bipolar disorder.^[11–13]^ The reason for the delayed diagnosis of DWM is not clear. Some suggest that a good communication between posterior fossa cyst membrane and the surrounding basal cisterns and distal pathways may explain the prolonged asymptomatic condition; the onset of symptoms in such cases may be attributed to age-related degeneration of the communicating channels.^[10]^ Again, as one of the most prominent pathological processes involved in DWM, hydrocephalus usually presents in the early years of disease onset, causing symptoms leading to the detection of DWM, which has already led to neurological damage. The aim of treatment is to prevent further damage caused by elevated intracranial pressure and compression caused by the enlarged ventricle. Shunting procedures have yielded satisfactory results in some patients; however, the choice of the precise procedure may vary from ventriculoperitoneal shunting to cystoperitoneal shunting and endoscopic third ventriculostomy, depending on the extent of obstruction of the aqueduct and subarachnoid space.^[14]^

Few studies have investigated the association between DWM and neoplastic disease. A literature search using the keywords “dandy-walker malformation,” “dandy-walker syndrome,” “dandy-walker variant,” “tumor,” “cancer,” and “neoplasm” retrieved 33 detailed studies (41 cases) of neoplasm concurrent with DMW (Table 1).^[15–47]^ Congenital vascular diseases were excluded as seen in PHACE syndrome. Out of the 41 cases, 8 patients had more than 1 neoplastic disease. The cases involved a variety of neoplasms including leukemia, teratoma, gastrointestinal stromal tumor, hamartoma, ganglioglioma, papillary thyroid carcinoma, retinoblastoma, and lipoma. Three cases had intracranial neoplasms (1 cerebellar teratoma, 1 hypothalamus ganglioglioma, and 1 hypothalamic hamartoma). In some of the cases, the underlying genetic or chromosomal cause was identified, while in the other cases, the etiology was unknown. Two most commonly reported neoplastic conditions in patients with DWM were neurocutaneous melanosis (16 cases, 39%), followed by urological neoplasms (13 cases, 31.7%) including Wilms tumor (8 cases), cystic nephroblastoma (3 cases), and rhabdomyosarcoma (2 cases).

**Table 1:** Previously reported cases of Dandy-Walker complex with concomitant neoplasms.

Ref no.	Author and year	Age and sex	Concurrent neoplasm	Confirmed chromosome or genetic alteration	Associated syndrome
^[15]^	Seok-Gu Kang, 2006	29 years, M	Neurocutaneous melanosis, intracranial meningeal melanocytoma, intracranial dermoid tumor	NA	NA
^[16]^	Tobias Walbert, 2009	25 years, F	Neurocutaneous melanosis, primary meningeal melanoma	NA	NA
^[17]^	M. Go¨ nu¨ l, 2009	30 years, F	Neurocutaneous melanosis, lipoma	NA	NA
^[18]^	F. Rodjan, 2010	1 year, M	Retinoblastoma	13q deletion syndrome	13q deletion syndrome
^[19]^	Kyung Hwan Kim, 2012	8 years, M	Neurocutaneous melanosis	NA	NA
^[20]^	Takayuki Azukizawa, 2013	1 year, F	Hypothalamic hamartoma		Oral-facial-digital syndrome type 1
^[21]^	Isabel Huguet, 2013	43 years, F	Papillary thyroid carcinoma, abdominal paraganglioma	(SDH) type D mutation (c.60_63delGCTT)	
^[22]^	Jens De Cock, 2014	Infant, M	Neurocutaneous melanocytosis	NA	NA
^[23]^	Julia Poschl, 2015	59 years, M	Cerebellar teratoma, lung carcinoma	NA	NA
^[24]^	N. Gupta, 2016	Fetus, M	Nasopharyngeal teratoma	variant (chr22:g.19371210C>T or NM_003325.3:c.1348G>A corresponding to p.V450I in the conserved HIRA B motif region) in HIRA	NA
^[25]^	Hideto Teranishi, 2018	3 years, F	Wilms tumor	Mutation of KMT2D; c.13285C>T: p.Q4429*	Kabuki syndrome
^[26]^	Rohit Kapoor, 2020	2 years, M	Acute promyelocytic leukemia	t(15;17), PMLRARA fusion gene and FLT3-ITD mutation	NA
^[27]^	Jose R. Infante, 2015	52 years, M	Gastrointestinal stromal tumor	NA	NA
^[28]^	David J Aughton, 1990	Infant, F	Nasopharyngeal teratoma	NA	NA
^[29]^	J. C. Chaloupka, 1996	Infant, M	Neurocutaneous melanosis	NA	NA
^[30]^	Randall D. Craver, 1995	1.5 years, F	Neurocutaneous melanosis, leptomeningeal melanoma	NA	NA
^[31]^	Lawrence J. Green, 1997	Infant, M	Neurocutaneous melanosis	NA	NA
^[32]^	Julie N. Kadonaga, 1992	Infant, M	Neurocutaneous melanosis	NA	NA
^[33]^	Hiroshi Kawame, 1999	1.6 years, M	Wilms tumor	Mosaicism of 48,XY,+7,+19[6]/46,XY[9]	Syndrome of microcephaly
^[34]^	Takashi Shuto, 1999	Infant, F	Spinal lipoma	NA	NA
^[35]^	Shinya Matsuura, 2000	Infant, M	Wilms tumor	Mosaic variegated aneuploidy syndrome	Mosaic variegated aneuploidy syndrome
^[35]^	Shinya Matsuura, 2000	Infant, M	Wilms tumor	Mosaic variegated aneuploidy syndrome	Mosaic variegated aneuploidy syndrome
^[36]^	Mena-Cedillos, 2002	5 years, M	Neurocutaneous melanosis	NA	NA
^[37]^	Yasuhiro Nakumura, 1984	1.3 years, F	Cystic nephroblastoma	46,XX/47,XX + 8 mosaicism	NA
^[38]^	T.Kinoshita, 1986	Infant, M	Cystic nephroblastoma, botryoid sarcoma	NA	NA
^[39]^	H.S. Narayanan*, 1987	2 years, M	Neurocutaneous melanosis	NA	NA
^[40]^	Zohreh Habibi, 2020	1 year, M	Neurocutaneous melanosis	NA	NA
^[40]^	Zohreh Habibi, 2020	1 year, F	Neurocutaneous melanosis	NA	NA
^[40]^	Zohreh Habibi, 2020	24 years, M	Neurocutaneous melanosis	NA	NA
^[40]^	Zohreh Habibi, 2020	8 years, M	Neurocutaneous melanosis	NA	NA
^[41]^	Lei-Ming Wang, 2019	6 years, M	Hypothalamus ganglioglioma	NA	NA
^[42]^	Kleebsabai Sanpakit, 2013	NA	Pediatric renal tumor	NA	NA
^[43]^	Noriyuki Akasaka, 2013	2 years, M	Wilms tumor	Mosaic variegated aneuploidy syndrome	Mosaic variegated aneuploidy syndrome
^[44]^	Masayuki Arai, 2004	Infant, M	Neurocutaneous melanosis	NA	NA
^[45]^	Toshiharu Furukawa, 2003	Infant, F	Rhabdomyosarcoma, nephroblastoma	Mosaic variegated aneuploidy syndrome	Mosaic variegated aneuploidy syndrome
^[46]^	Tadashi Kajii, 2001	Infant, M	Wilms tumor	Mosaic variegated aneuploidy syndrome	Mosaic variegated aneuploidy syndrome
^[46]^	Tadashi Kajii, 2001	Infant, F	Wilms tumor	Mosaic variegated aneuploidy syndrome	Mosaic variegated aneuploidy syndrome
^[46]^	Tadashi Kajii, 2001	Infant, F	Wilms tumor	Mosaic variegated aneuploidy syndrome	Mosaic variegated aneuploidy syndrome
^[46]^	Tadashi Kajii, 2001	Infant, M	Botryoid rhabdomyosarcoma	Mosaic variegated aneuploidy syndrome	Mosaic variegated aneuploidy syndrome
^[46]^	Tadashi Kajii, 2001	Infant, M	Polycystic nephroblastoma	Mosaic variegated aneuploidy syndrome	Mosaic variegated aneuploidy syndrome
^[47]^	Riitta Herva, 1996	4.5 years, M	Botryoid rhabdomyosarcoma	NA	NA

Neurocutaneous melanosis is a rare congenital syndrome characterized by the presence of large or multiple congenital melanocytic nevi and benign or malignant pigment cell tumors of the leptomeninges, possibly resulting from mutations of the *NRAS* and *BRAF* gene.^[48]^ The commonly discussed mechanisms for the association between neurocutaneous melanosis and DWM are obstruction by melanocytes of the outgoing foramen of the 4th ventricle; developmental error of the cerebellum and 4th ventricle induced by the leptomeningeal abnormalities; and the interference by leptomeningeal melanocytosis with the primitive meningeal cells.^[49]^ Literature review performed in our study indicates the concurrence of these 2 conditions in newborns, pediatric patients, as well as in adult patients; in addition, we observed a predilection for the male sex (male:female ratio 3:1).

In contrast, the association between DWM and urological neoplasms was exclusively observed in newborns, the majority of whom carried chromosomal alterations. The most frequently detected condition in these patients was the Mosaic variegated aneuploidy syndrome, which is a rare cancer-prone disorder associated with an autosomal-recessive trait related to *BUB1B* gene mutations.^[50]^ The severity of Mosaic variegated aneuploidy syndrome may vary from mild clinical symptoms (such as only growth retardation) to the concomitant occurrence of DWM and pediatric malignancies such as Wilms tumor (most severe form). The reason for the development of DWM in Mosaic variegated aneuploidy syndrome remains unknown. A potential explanation is the alteration of *BUB1B* function contributing to abnormal infratentorial development.^[43]^ Our literature review revealed a higher predilection for male sex (male:female ratio 2.25:1).

The concurrence of DWM with PXA has not been documented before. PXA belongs to a rare subgroup of astrocytic tumors first described in 1979 by Kepes.^[51]^ The imaging features of PXA mimic those of high-grade gliomas; however, the majority of PXAs affect young adults, and the mean age of disease onset for PXA is 29 years.^[2]^ Even though the PXAs share some imaging features of high-grade gliomas, the disease course is usually long and insidious and the condition is associated with a relatively good prognosis following complete surgical resection. The most common site of occurrence of PXA is the temporal lobe, usually associated with chronic epilepsy due to its tendency for gyral involvement. Cases involving deep location without gyral involvement are very rare, as in the present case. Three similar cases have been reported: a 28-year-old man with incidental finding of a left lateral ventricular PXA^[52]^; a 52-year-old man with headache and right lateral ventricular PXA^[53]^; and a 42-year-old woman with right lateral ventricle PXA that underwent subarachnoid dissemination.^[54]^ Our patient had a previous history of surgery for the removal of lateral ventricle meningioma. However, we were unable to gain access to the previous medical records; hence, we were unable to confirm whether the current ventricular PXA was a primary lesion after meningioma resection or a local recurrence after the last surgery for the lesion which was actually a PXA instead of a meningioma. The pathogenesis of PXA, as well as other gliomas, remains largely unknown. PXAs are usually devoid of many molecular patterns shared by other WHO grade II tumors, such as *TP53* mutation or *EGFR* mutation overexpression, but have more similarities, including *BRAF* mutations, with WHO grade I pilocytic astrocytomas, which also have a tendency to occur earlier in life.^[55]^ Compared to common cerebral neoplasms occurring later in life which have a multifactorial etiology, congenital genetic factors may play a more prominent role in the pathogenesis of PXAs. A literature review identified reports of PXA concurrent with congenital diseases including Sturge-Weber syndrome,^[56]^ constitutional 22q11.2 deletion syndrome,^[57]^ Familial melanoma-astrocytoma syndrome,^[58]^ and Down syndrome.^[59]^ Interestingly, as one of the most common mutations found in PXAs, *BRAF* V600E mutation has also been reported to be associated with neurocutaneous melanosis,^[48]^ which could imply an underlying indirect link between PXA and DWM in our case.

In conclusion, the association between Dandy-Walker complex and various neoplasms may be due to underlying chromosomal alterations and genetic mutations or may be incidental. We report the first case of PXA concurrent with Dandy-Walker complex in a young adult. This very rare association could be more than a rare coincidence and may imply a hidden link between these 2 rare disease entities and possibly other congenital diseases such as neurocutaneous melanosis. Future studies related to these conditions may help characterize the association between embryonic development and oncogenesis, especially in the central nervous system.

## Author contributions

T-FL contributed to the writing of the original draft. Y-BW contributed to the methodology and data curation. D-HW contributed to the pathology analysis. SZ contributed to the imaging analysis. S-LD contributed to the conceptualization and review & editing.

**Conceptualization:** Shuang-Lin Deng.

**Data curation:** Yu-Bo Wang.

**Formal analysis:** Dan-Hua Wang, Shuang Zhan.

**Methodology:** Yu-bo Wang.

**Writing – original draft:** Tian-Fei Luo.

**Writing – review & editing:** Shuang-Lin Deng.
